# Adverse Renal Outcomes in Patients With Mesothelioma—A Territory‐Wide Real‐World Data

**DOI:** 10.1002/cam4.71595

**Published:** 2026-01-30

**Authors:** Wang Chun Kwok, James Chung Man Ho, Isaac Sze Him Leung, Desmond Yat Hin Yap

**Affiliations:** ^1^ Division of Respiratory Medicine, Department of Medicine, School of Clinical Medicine, LKS Faculty of Medicine The University of Hong Kong Pok Fu Lam Hong Kong; ^2^ Department of Medicine Queen Mary Hospital Pok Fu Lam Hong Kong; ^3^ Department of Statistics The Chinese University of Hong Kong Shatin, New Territories Hong Kong SAR China; ^4^ Division of Nephrology, Department of Medicine, School of Clinical Medicine, LKS Faculty of Medicine The University of Hong Kong Pok Fu Lam Hong Kong

**Keywords:** acute kidney injury, chronic kidney disease, mesothelioma, renal progression, risk factors

## Abstract

**Introduction:**

Advances in mesothelioma management have translated into longer patient survival and different treatment‐related side effects including nephrotoxicity. The risk of developing adverse renal outcomes in patients with mesothelioma and associated risk factors remains undefined.

**Methods:**

We analysed territory‐wide data from electronic health records of patients with mesothelioma followed at public hospitals in Hong Kong between 1st January 2000 to 31st December 2022. Prevalence of acute kidney injury (AKI), renal progression (> 30 mL/min drop in eGFR), and upstaging of chronic kidney disease (CKD) and associated risk factors were evaluated.

**Results:**

222 patients were included. 18 (5.1%) patients developed acute kidney injury (AKI), and risk factors included diabetes mellitus (DM), use of bevacizumab and the presence of third space fluid (pleural effusion, pericardial effusion, ascites). 47 (21.2%) patients had upstage of CKD, and 31 (14.0%) patients showed renal progression. 18, 9, and 4 patients developed renal progression within 12 months from diagnosis, 12–24 months from diagnosis, and more than 24 months from diagnosis. Risk factors for upstage of CKD included the presence of third space fluid, platinum‐based chemotherapy, use of immune check‐point inhibitors, AKI during follow‐up, more lines of cytotoxic chemotherapy received, and cycles of pemetrexed used. Predictors for renal progression included the presence of ascites and use of bevacizumab.

**Conclusion:**

Short‐ and long‐term adverse kidney outcomes are prevalent in patients with mesothelioma and show strong associations with treatments received. Careful patient selection and close monitoring of renal function may help avoid untoward acute and chronic nephrotoxicity.

## 1 Background

Mesothelioma is an uncommon neoplasm arising from mesothelial surfaces of the pleural cavity, peritoneal cavity, tunica vaginalis, or pericardium that is associated and malignant pleural mesothelioma (MPM) is the commonest among all subtypes, accounting for 80% of the cases [1].

The management of MPM has evolved substantially over the past few decades, adopting a multidisciplinary treatment plan based upon the assessment of disease extent, the patient's functional state and concomitant medical comorbidities, as well as their desire for aggressive therapies. Approximately 20% of the patients may be suitable for surgery with a macroscopic complete resection as part of a combined‐modality approach [2]. For patients who are not surgical candidates, they receive standard treatments with chemotherapy comprising pemetrexed and platinum or nivolumab/ipilimumab as approved by US Food and Drug Administration (FDA) and European Medicines Agency (EMA) [3, 4, 5, 6, 7, 8]. For pemetrexed, after an initial four to six cycles of platinum‐based doublet, the use of maintenance treatment does not have enough evidence to support its use despite some small‐scale studies suggesting its benefits [9, 10] while other studies did not [11]. Bevacizumab can be added to chemotherapy that was shown to have prolonged progression‐free survival and overall survival [12]. Nivolumab plus ipilimumab has been shown to provide long‐term survival benefits over chemotherapy with a manageable safety profile, especially among patients with non‐epithelioid histology [13].

Advances in mesothelioma management have translated into improved patient survival and at the same time treatment‐related side effects including nephrotoxicity. For pemetrexed, the presence of non‐evacuated third‐space fluid during treatment course and receiving more than 15 cycles of maintenance pemetrexed were identified as independent risk factors to the development of nephrotoxicity associated with maintenance pemetrexed use in metastatic non‐squamous NSCLC [14]. This is of particular relevance to mesothelioma as the presence of pleural effusion, pericardial effusion and ascites are common. On the other hand, platinum‐based chemotherapy is also a well‐recognized treatment with nephrotoxic potential [15, 16, 17]. Bevacizumab, as an anti‐angiogenic, is also reported to be associated with proteinuria, nephrotic syndrome [18, 19, 20, 21, 22], nephritic syndrome, acute kidney injury, and proliferative glomerulonephritis [23]. For example, pemetrexed is contraindicated in patients with creatinine clearance below 45 mL/min [24]. The presence of acute kidney injury or chronic kidney disease in patients with mesothelioma has important clinical implications, as this may predispose them to a higher risk of treatment‐associated complications and also limit the choice of therapies. Furthermore, cancer patients with kidney failure showed significantly increased risk of mortality compared to those without [25, 26]. However, there is limited data on adverse renal outcomes in patients with mesothelioma in the current era and the predictors of unfavorable kidney outlook remain undefined. In this study, we used territory‐wide data to investigate the burden of adverse kidney outcomes in patients with mesothelioma and identify important risk factors. The findings will help patient selection for appropriate cancer therapeutics and hence improve clinical outcomes and cost‐effectiveness of treatment.

## 2 Methods

Patients with mesothelioma managed and followed at public hospitals under the Hong Kong Hospital Authority (HKHA) between 1st January 2000 to 31st December 2022 were included for analysis. The HKHA is the statutory body that operates all public hospitals (43 hospitals and institutions) and outpatient clinics (122 clinics) in Hong Kong. Study populations were identified through the Clinical Data Analysis and Reporting System (CDARS), which is an electronic healthcare database managed by HKHA covering 90% of healthcare services in Hong Kong.

Patients aged 18 or above with the diagnostic coding of mesothelioma were included. Patients who had another malignancy before or after diagnosis of mesothelioma, with incomplete baseline clinical details of investigation results, lost to follow up, without treatment record, incomplete investigation results upon follow‐up and with the diagnostic code but labeled as provisional diagnosis only or labeled as “history of mesothelioma” which could suggest the development of the mesothelioma before the inclusion period were excluded. Demographic data (including age, sex, diagnosis of mesothelioma and other comorbidities, therapy for mesothelioma, renal function by estimated glomerular filtration rate (eGFR) as calculated by Chronic Kidney Disease Epidemiology Collaboration (CKD‐EPI) equation), clinical outcome (acute kidney injury, development of renal progression, upstage in chronic kidney disease stage) were collected through CDARS.

The co‐primary outcomes included the prevalence of adverse renal outcomes (AKI, development of renal progression, upstage in chronic kidney disease stage) and the risk factors to predict their development. AKI is defined as an increase in serum creatinine (Cr) by ≥ 26.5 μmol/L within 48 h, or an increase in serum Cr to ≥ 1.5 times baseline, which is known or presumed to have occurred within the prior 7 days [27]. The stage of AKI by KDIGO criteria is defined as follows: Stage 1: Increase in serum creatinine (SCr) ≥ 0.3 mg/dL (26.52 μmol/L) (in 48 h) or 1.5–1.9 times of baseline values (within 7 days); Stage 2: 2.0–2.9 times of baseline SCr; Stage 3: ≥ 3.0 times baseline; increase in SCr ≥ 4.0 mg/dL; or the beginning of renal replacement therapy regardless of a previous KDIGO stage. Renal progression was defined as a decrease in estimated glomerular filtration rate (eGFR) of > 30 mL/min/1.73 m^2^ [28]. Upstage in CKD stage was defined as an increase in one or more CKD stages as defined by K/DOQI clinical practice guidelines for CKD: evaluation, classification, and stratification definition [28]. This study was approved by The University of Hong Kong and Hospital Authority Hong Kong West Cluster Institutional Review Board (approval reference number: UW 24‐126). Patients' informed consent was waived by the Institutional Review Board as this is a retrospective study without active subject recruitment.

## 3 Statistical Analysis

The demographic and clinical data were described in actual frequency or mean ± standard deviation (SD), or median (25th to 75th centile) where appropriate. Baseline demographic and clinical data were compared between patients with or without adverse renal outcomes by independent *t*‐test or nonparametric tests where appropriate. Multiple logistic regression modeling was also used to take into account covariates (age, sex, year of diagnosis, baseline eGFR, Charlson Comorbidity Index (CCI), underlying hypertension, diabetes mellitus, and lines of chemotherapy used). These covariates were chosen as they were well reported risk factors of adverse renal outcomes related to cancer treatment [29, 30, 31] or as a standalone condition linked to renal impairment (such as hypertension and diabetes mellitus). Subgroup analyses were performed for patients who were treated with National Comprehensive Cancer Network (NCCN) recommended treatment. Multicollinearity of the covariates was tested and summarized in Table S1. For covariates with multicollinearity, they would be analysed separately. Statistical significance was determined at the level of *p* < 0.05 (2‐sided test). All statistical analyses were done using the 28th version of SPSS statistical package.

## 4 Results

### 4.1 Patient Characteristics

**FIGURE 1:**
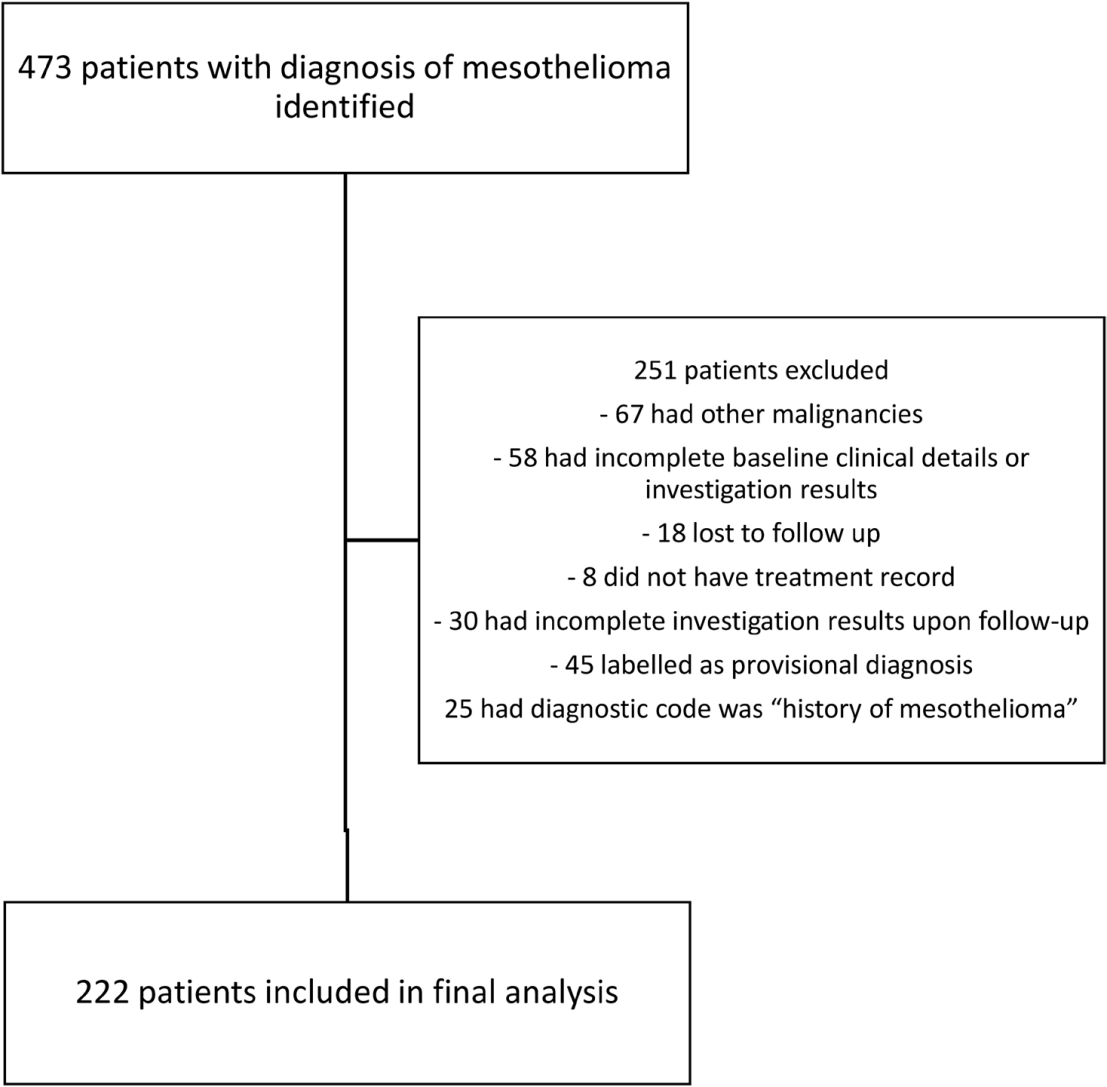
Patient selection flow diagram.

There were 473 patients who had the diagnosis of mesothelioma identified in CDARS. 251 patients were excluded as they met the exclusion criteria: 67 had a history of other malignancies or the presence of other malignancies during the follow‐up period; 58 had incomplete baseline clinical details or investigation results; 18 lost to follow up; 8 did not have treatment record (which affected the primary outcome); 30 had incomplete investigation results upon follow‐up; 45 had the diagnostic code but labelled as provisional diagnosis only and 25 excluded as the diagnostic code was “history of mesothelioma” which could suggest the development of the mesothelioma before the inclusion period. The patient selection flow‐diagram was illustrated in Figure 1. Among the 222 patients included in the final analysis (Table 1), the mean age was 54.2 ± 14.8 years. 152 (68.5%) of the patients were male. The median follow‐up duration was 1.28 [0.55–2.87] years. The median baseline Charlson co‐morbidity index was 8 (7–9). The baseline serum eGFR was 110.0 ± 33.5 mL/min/1.73 m^2^ Patients with or without adverse renal outcomes (AKI, renal progression or upstage in CKD) were compared with the baseline demographics summarized in Table 1.

**TABLE 1:** Baseline demographic and clinical characteristics.

	Whole cohort (*n* = 222)	Adverse renal outcomes (*n* = 62)	No adverse renal outcomes (*n* = 160)	*p*
Age (years), mean ± SD	54.2 ± 14.8	54.6 ± 15.6	54.1 ± 14.5	0.85
Male (%)	152 (68.5%)	42 (67.7%)	110 (68.8%)	0.89
Year of diagnosis				
2000–2005	11 (5.0%)	3 (4.8%)	8 (5.0%)	< 0.001
2006–2010	68 (30.6%)	13 (21.0%)	55 (34.4%)	
2011–2015	56 (25.2%)	6 (9.7%)	50 (31.3%)	
2016–2022	87 (39.2%)	40 (64.5%)	47 (29.4%)	
Baseline eGFR (mL/min/1.73 m^2^), mean ± SD	110.0 ± 33.5	115.0 ± 35.4	106.3 ± 31.8	0.13
Co‐morbidities (%)				
Hypertension	41 (18.5%)	10 (16.1%)	31 (19.4%)	0.58
Diabetes mellitus	17 (7.7%)	9 (14.5%)	8 (5.0%)	0.02*
Ischaemic heart disease	13 (5.9%)	0 (0%)	13 (8.1%)	0.05
Heart failure	12 (5.4%)	0 (0%)	12 (7.5%)	0.06
COPD	15 (6.8%)	5 (8.1%)	10 (6.3%)	0.63
Old stroke	10 (4.5%)	4 (6.5%)	6 (3.8%)	0.38
Charlson co‐morbidity index, median (25th–75th centile)	8 (7–9)	8 (7–10)	8 (7–9)	0.14
Disease involvement (%)				
Pleural effusion	105 (47.3%)	32 (51.6%)	73 (45.6%)	0.42
Pericardial effusion	18 (8.1%)	2 (3.2%)	16 (10.0%)	0.10
Ascites	16 (7.2%)	8 (12.9%)	8 (5.0%)	0.04*
Brain metastasis	14 (6.3%)	3 (4.8%)	11 (6.9%)	0.58
Bone metastasis	26 (11.7%)	7 (11.3%)	19 (11.9%)	0.90
Treatment received (%)				
Pemetrexed	100 (44.5%)	27 (43.5%)	73 (45.6%)	0.78
Gemcitabine	53 (23.9%)	25 (40.3%)	28 (17.5%)	< 0.001*
Carboplatin	70 (31.5%)	25 (40.3%)	45 (28.1%)	0.08
Cisplatin	44 (19.8%)	11 (17.7%)	33 (20.6%)	0.63
Mitomycin C, vinblastine and cisplatin/carboplatin (MVP)	20 (9.0%)	8 (12.9%)	12 (7.5%)	0.89
Doxorubicin	89 (40.1%)	29 (46.8%)	60 (37.5%)	0.21
5‐fluorouracil	4 (1.8%)	0 (0%)	4 (2.5%)	0.21
Epirubicin	8 (3.6%)	1 (1.6%)	7 (4.4%)	0.32
Vinorelbine	2 (0.9%)	0 (0%)	2 (1.3%)	0.38
Etoposide	23 (10.4%)	10 (16.1%)	13 (8.1%)	0.08
Methotrexate	5 (2.3%)	1 (1.6%)	4 (2.5%)	0.69
Bevacizumab	9 (4.1%)	8 (12.9%)	1 (0.6%)	< 0.001*
Immune check‐point inhibitors	11 (5.0%)	9 (14.5%)	2 (1.3%)	< 0.001*
Number of lines of chemotherapy				
1	95 (42.8%)	20 (32.3%)	75 (46.9%)	0.03*
2	87 (39.2%)	24 (38.7%)	63 (39.4%)	
≥ 3	40 (18.1%)	18 (29.0%)	20 (13.7%)	
Number of cycles of pemetrexed, median [25th–75th centile]	4 [4–6]	4 [4–12]	4 [4–4]	0.07

* Statistical significance.

### 4.2 AKI and Mesothelioma

Eighteen (8.1%) patients developed AKI. Among the patients who developed AKI, 9 had stage 1 AKI, 7 had stage 2 AKI, and 2 had stage 3 AKI. Among the patients who developed AKI, 7 (38.9%) progressed to CKD, while 24 (11.8%) of the patients who did not experience AKI progressed to CKD. 11 (61.1%) of the patients had normalization of renal function after the AKI episode. The risk factors for AKI included the presence of diabetes mellitus (adjusted odds ratios (aOR) 23.13, 95% CI 1.96–272.62, *p* = 0.013), the use of bevacizumab (aOR 61.67, 95% CI 2.01–1896.05, *p* = 0.018), and the presence of third space fluid (aOR 8.19, 95% CI 1.23–54.33, *p* = 0.029) (Table 2).

**TABLE 2:** Risk factors for adverse renal outcomes.

	OR	95% CI	*p*	aOR^a^	95% CI	*p*
Renal progression						
Ascites	3.15	1.01–9.78	0.048	4.07	0.92–18.05	0.07
Bevacizumab	15.04	3.53–63.94	< 0.001	13.19	1.81–96.19	0.011*
Upstage of CKD						
Third space fluid	1.87	0.97–3.61	0.06	2.25	0.99–5.07	0.052
Ascites	3.23	1.13–9.19	0.028	9.40	1.84–48.07	0.007*
Platinum	1.61	0.84–3.08	0.15	5.57	2.03–15.30	< 0.001
ICI	20.49	4.25–98.66	< 0.001	28.81	2.94–282.27	0.004*
AKI	13.00	4.44–38.87	< 0.001	9.84	2.38–40.76	0.002*
Number of lines of cytotoxic chemotherapy	1.73	1.19–2.51	0.004	2.11	1.31–3.40	< 0.001*
Number of pemetrexed cycles	1.03	1.00–1.06	0.042	1.06	1.01–1.10	0.011*
AKI						
DM	6.15	1.88–20.13	0.003	23.13	1.96–272.62	0.013*
Bevacizumab	6.60	1.50–29.05	0.013	61.67	2.01–1896.05	0.018*
Third space fluid	3.94	1.25–12.37	0.019	8.19	1.23–54.33	0.029*
Pleural effusion	3.17	1.09–9.21	0.034	5.57	0.90–34.30	0.06
Number of pemetrexed cycles	1.05	1.01–1.08	0.008	1.02	0.96–1.09	0.53
Adverse renal outcomes						
DM	3.23	1.18–8.79	0.022	7.60	1.57–36.80	0.012*
Ascites	2.82	1.01–7.87	0.048	8.27	1.41–48.66	0.019*
Gemcitabine	3.19	1.66–6.11	< 0.001	1.93	0.69–5.42	0.21
ICI	13.42	2.81–64.06	0.001	18.23	2.00–165.93	0.010*
Bevacizumab	23.56	2.88–192.68	0.003	24.80	2.54–242.08	0.006*
Number of lines of cytotoxic chemotherapy	1.76	1.24–2.50	0.002	2.30	1.43–3.69	< 0.001*

Abbreviations: AKI, acute kidney injury; aOR, adjusted odds ratios; CI, confidence interval; DM, diabetes mellitus; ICI, immune check‐point inhibitors; OR, Odds ratios.

^a^ Adjusted for age, sex, year of diagnosis, presence of hypertension, diabetes mellitus, baseline estimated glomerular filtration rate, lines of chemotherapy, and Charlson co‐morbidity index.

* Statistically significant.

The mean time from initiation of bevacizumab to onset of AKI was 4.35 (95% CI = 3.34–5.26) months.

### 4.3 Renal Progression and Upstage in CKD in Patients With Mesothelioma

There were 183 (82.4%), 38 (17.1%), and 1 (0.5%) patients with stage 1, 2, and 3 CKD at the time of diagnosis, and the corresponding number and rates were 144 (64.9%), 67 (30.2%), and 11 (5%) at the end of the study period. 47 (21.2%) patients had upstage of CKD. The risk factors for upstage in CKD included the presence of ascites (aOR 9.40, 95% CI 1.84–48.07, *p* = 0.007), the use of platinum (aOR 5.57, 95% CI 2.03–15.30, *p* < 0.001), the use of ICI (aOR 28.81, 95% CI 2.94–282.27, *p* = 0.004), the development of AKI during the follow‐up period (aOR 9.84, 95% CI 2.38–40.76, *p* = 0.002), the number of lines of cytotoxic chemotherapy received (aOR 2.11, 95% CI 1.31–3.40, *p* < 0.001), and the number of pemetrexed cycles used (aOR 1.06, 95% CI 1.01–1.10, *p* = 0.011) (Table 2).

There were 31 (14.0%) patients who developed renal progression in the follow‐up period, which was defined as a decrease in eGFR of more than 30 mL/min/1.73 m^2^. 18, 9, and 4 patients developed renal progression within 12 months from diagnosis, 12–24 months from diagnosis and more than 24 months from diagnosis. None of the patients developed end‐stage kidney disease. The risk factors for renal progression included the use of bevacizumab with aOR of 13.19 (95% CI 1.81–96.19, *p* = 0.011).

### 4.4 Adverse Renal Outcomes (AKI, Renal Progression or Upstage in CKD)

There were 62 (27.9%) patients who developed adverse renal outcomes (AKI, renal progression and upstage in CKD) in the follow‐up period. The risk factors for adverse renal outcomes included the presence of diabetes mellitus (aOR 7.60, 95% CI 1.57–36.80, *p* = 0.012), the presence of ascites (aOR 8.27, 95% CI 1.41–48.66, *p* = 0.019), the use of bevacizumab (aOR 24.80, 95% CI 2.54–242.08, *p* = 0.006), the use of ICI (aOR 18.23, 95% CI 2.00–165.93, *p* = 0.010), and the number of lines of cytotoxic chemotherapy received (aOR 2.30, 95% CI 1.43–3.69, *p* < 0.001) (Table 2).

### 4.5 Subgroup Analysis

Subgroup analysis was performed among those patients treated with NCCN recommended treatment (pemetrexed, gemcitabine, cisplatin or carboplatin, pembrolizumab, nivolumab, ipilimumab, bevacizumab, vinorelbine). There were 161 subjects in this subgroup. The results in the subgroup analysis were largely consistent with the analysis in the whole cohort, with the results summarized in Table S2.

The risks for adverse renal outcomes among patients who received cisplatin and carboplatin were compared, which did not show a significant difference with *p* > 0.05 for all the outcomes.

## 5 Discussion

Our study demonstrated that adverse renal outcomes from mesothelioma treatment are common. While some of these adverse events are transient, some of them can accumulate or gradually progress, leading to long‐term adverse outcomes.

With the advancement in treatment for mesothelioma, we are expecting that the patients to have longer survival with more treatment choices. This will be translated to receiving more therapeutic agents including cytotoxic chemotherapy, immunotherapy and anti‐VEGF. In our cohort, over one‐fifth had upstaging of CKD at the end of the study. Here we observed that the more the lines of cytotoxic chemotherapy, the higher the risks of CKD upstage, which is consistent with the accumulated toxicity of cytotoxic chemotherapy. First of all, exposure to different nephrotoxic chemotherapy over time could lead to adverse renal outcomes. Secondly, cumulative doses of individual nephrotoxic agents (such as cisplatin and pemetrexed) were also reported as risk factors for adverse renal outcomes [32, 33]. The development of CKD has important implications on subsequent patient management, including the choice of future therapeutic agents, dosage adjustment and risks of side effects and complications of associated treatments [34]. While CKD appeared to be rather common in survivors of mesothelioma after receiving standard treatments, the incidence of AKI was relatively low. The low incidence of AKI may be related to the relatively good baseline renal function and also appropriate dosages of platinum‐based treatments. Furthermore, most cases were mild AKI and over 60% had renal recovery.

The risk of CKD was strongly associated with disease extent and treatment history. As a malignancy arising from the serosal surface, mesothelioma has a tendency to develop third‐space fluid. At the same time, pemetrexed was also the cytotoxic chemotherapy that has a tendency to accumulate in third‐space fluid. These two factors that were reported to be risk factors of adverse renal outcomes may interact with each other and result in adverse renal outcomes, as in NSCLC. Similar findings were first reported in methotrexate, which has pharmacological properties similar to pemetrexed, being with low plasma protein binding. The presence of third‐space fluid will result in elevated serum methotrexate levels at 48–72 h post‐infusion and also increase the risk of AKI [35]. Similar findings were also reported in NCLSC, with the presence of non‐evacuated third‐space fluid during treatment course to be the risk factor for development of nephrotoxicity in NSCLC patient receiving first‐line pemetrexed‐platinum doublets [14]. Pemetrexed is one of the most commonly used cytotoxic chemotherapies in mesothelioma as in NSCLC, and it has been well reported that hydrophilic drugs, particularly those with low plasma protein binding (e.g., pemetrexed and methotrexate used in the treatment of mesothelioma), may accumulate in third‐space fluid in the body [36]. Prior studies suggested that the presence of non‐evacuated third‐space fluid in patients with advanced NSCLC can predispose patients receiving pemetrexed and platinum chemotherapy to significant hematological toxicity and nephrotoxicity [14, 37]. While drainage of third‐space fluid may relieve symptoms and potentially reduce the risk of treatment‐related nephrotoxicity in patients with mesothelioma, such clinical decisions should be undertaken with caution, as more than a quarter of patients with mesothelioma who had indwelling pleural catheters (IPCs) for the management of symptomatic pleural effusions developed catheter tract metastasis [38]. Routine delivery of prophylactic radiotherapy to prevent procedure tract metastases in mesothelioma was not recommended, as the study demonstrated disappointing results [39]. Instead, palliative radiotherapy in case procedure tract metastases developed could be considered [39]. The decision on the drainage of pleural effusion and other third‐space fluid shall involve detailed discussion on the pros and cons between clinicians and patients. The volume of drainage should be carefully monitored, and intravascular volume depletion should be corrected to mitigate the risk of kidney injury. Alternative approaches such as chemical pleurodesis should also be considered when the patient had initial therapeutic drainage, which involves a shorter duration of drainage catheter in place.

Our data also suggested that the risk of CKD was associated with the type of treatment (e.g., platinum chemotherapy and ICI) and the cumulative toxicities (number of lines of treatment and cycles of pemetrexed). Immunotherapy such as immune check‐point inhibitors and their associations with tubulointerstitial nephritis and glomerulonephritis are also important causes of treatment‐related nephrotoxicity. The overall incidence of immune check‐point inhibitors associated AKI is approximately 1.5%–5% [40, 41] with acute tubulointerstitial nephritis being the most common pathology, while immune complex glomerulonephritis and thrombotic microangiopathy were also observed [42, 43, 44]. Although some suggested that immunotherapy can be rechallenged upon recovery from AKI [44], whether this can contribute to long‐term renal outcomes needs to be properly assessed as a small subgroup of patients will experience recurrent AKI upon re‐challenge of immunotherapy [44]. Hence, the decision of re‐challenge with immunotherapy should be carefully considered.

Another drug that was suggested with adverse renal outcomes was bevacizumab, which was reported to be associated with chronic renal impairment and thrombotic microangiopathy [45]. While thrombotic microangiopathy could explain AKI, long‐term renal outcome can result from resolved AKI, worsening hypertension, proteinuria, chronic kidney disease, and glomerular disease [45]. Based on the knowledge of nephrotoxicity from bevacizumab, routine monitoring of blood pressure, urine protein, and renal function shall be needed among those treated with bevacizumab. Proper blood pressure control should not be over‐emphasized as well. For diabetes mellitus, which was shown to be a risk factor for AKI and adverse renal outcomes in this study, the same phenomenon was also reported in other cancer types [46]. As such, it is important to have proper glycaemic control and monitoring for these patients during the treatment course, with regular renal function monitoring.

Of note, maintenance pemetrexed is not recommended by guidelines in treating mesothelioma, as there were no survival benefits demonstrated. CALGB 39091 study suggested that maintenance pemetrexed following initial pemetrexed and platinum chemotherapy did not improve progression‐free survival in patients with MPM [11], while haematological adverse events being the concern of maintenance pemetrexed. However, a small amount of the patients in this cohort were treated with maintenance pemetrexed as other studies suggested possible benefits [9, 10] while the treatment choices are limited for mesothelioma, especially when immunotherapy and bevacizumab are not covered by the current funding scheme of patients managed in a public healthcare setting. This may make the clinicians to be more willing to continue on pemetrexed which is a drug that the patient can receive for free in the short of other affordable alternatives. But the development of adverse events should not be forgotten, as we demonstrated that the number of cycles of pemetrexed received to be risk factors for upstage of CKD and AKI. Hence, the decision of maintenance pemetrexed shall be carefully decided. Among patients at increased risk of adverse renal outcomes, maintenance pemetrexed shall be avoided. Platinum use was also reported to be a risk factor of CKD upstage in the current study, while the risks of various adverse renal outcomes were not statistically different for cisplatin and carboplatin in this study. As immune checkpoint inhibitors were also recognized to be a treatment for mesothelioma, though the adverse renal outcomes from immune checkpoint inhibitors should not be forgotten as well.

Our study has several limitations to address. Firstly, the majority of the included patients were Chinese, and it may affect the generalizability of the study. But the treatment regime for mesothelioma is largely consistent among different ethnic groups, though ethnic differences in the pharmacokinetics could still be possible. A multination study that includes patients from different ethnic groups will be worthwhile to confirm the study findings presented here. Secondly, we excluded the 251 patients who met the exclusion criteria. This leads to a smaller sample size in this study. The drawback is that some risk factors for the adverse events may not be able to be demonstrated due to lack of statistical power. But it also has the benefit of including a more well‐defined patient cohort in this study by excluding the potentially inappropriate cases. Using CDARS allows us to have a gross assessment of the adverse events and the associated risk factors but also lacks the granular data. This may have minor limitations to this study as some patients' characteristics such as smoking and drinking history could not be properly assessed, though their impact on this study overall is minimal. Our study covers a prolonged period of more than 20 years with different treatment regimes covered in this period. This could explain that different treatment regimens were included in this study. Less than half of the patients were treated with pemetrexed, as it was only listed in the Hospital Authority formulary as a self‐financed item in 2007, included in the Samaritan fund for applications with financial subsidy in eligible patients in 2015, and eventually available as a special drug for free for all patients in 2019. This could explain the low usage rate of pemetrexed in this study due to availability issues. The detailed pathological details such as anatomical subtype (such as pleural or peritoneal mesothelioma), histological subtype, and detailed staging were not available in this study that utilized electronic health record. The absence of these details may limit the findings that focus on the treatment response and prognosis, but the impact on treatment‐related adverse events is less prominent, as the risk factors for adverse renal outcomes identified in this study were more related to the co‐morbidities and treatment details. The renal outcomes assessed in this study including AKI were defined using eGFR/serum creatinine, which is the most commonly used parameter [47, 48, 49, 50]. However, some other definitions also include other parameters such as urine output while other parameters may also be altered in AKI and other renal outcomes, such as urine protein, which were not available in this study. Lacking these parameters may underestimate the incidence of these renal outcomes to some degree and also make the differentiation of the aetiology of renal outcomes not possible. But these markers were not universally available and also may not be routinely repeated in the course of disease. It is also rare to have AKI or renal outcomes to develop without a change in eGFR/serum creatinine. Hence, lacking these data does not have a major impact on the study outcomes. As a rare malignancy, the sample size is limited even including patients treated for over 20 years. This resulted in a small event number and hence wide CI for some of the results which need careful interpretation. To address this issue, a larger dataset involving multi‐centres shall be considered, allowing a more precise assessment of rare events. While multicollinearity was identified with third space fluid and pleural effusion, it is rather expected and they were separately analysed in this study.

## 6 Conclusion

Adverse renal outcomes are common in mesothelioma treatment. Distinct risk factors related to underlying disease and treatment are pertinent to renal progression, upstaging of CKD and AKI. Among patients with high risks of adverse renal outcomes, appropriate measures such as optimizing diabetic control, evacuation of third space fluid and limiting the dose and cycles of platinum may be considered. As a malignancy arising from serosa, the risk factors identified in this study have clinical relevance given the fact that third space fluid tends to be present in mesothelioma while pemetrexed, which is one of the guidelines approved therapy, had the tendency to accumulate in the third space.

## Author Contributions

**Wang Chun Kwok:** conceptualization (equal), data curation (equal), formal analysis (equal), writing – original draft (equal), writing – review and editing (equal). **James Chung Man Ho:** writing – review and editing (equal). **Isaac Sze Him Leung:** writing – review and editing (equal). **Desmond Yat Hin Yap:** conceptualization (equal), supervision (equal), writing – original draft (equal), writing – review and editing (equal).

## Funding

This work was supported by Pneumoconiosis Compensation Fund Board (PCFB) Research Fund.

## Disclosure

Declaration of Generative AI and AI‐Assisted Technologies in the Writing Process: During the preparation of this work the authors did not use any AI tools/service.

## Ethics Statement

The study was approved by the Institutional Review Board of the University of Hong Kong and Hospital Authority Hong Kong West Cluster (UW 24‐126). Informed consents were waived as it was a retrospective study without active patient recruitment, and all retrieved clinical data were de‐identified.

## Conflicts of Interest

The authors declare no conflicts of interest.

## Supporting information


**Table S1:** Multicollinearity testing of the covariates.
**Table S2:** Risk factors for adverse renal outcomes among patients treated with NCCN recommended treatment.

## Data Availability

Dataset supporting the conclusion of this article is included within this article and no additional data will be provided. Research data is not shared.
