# Supplementary material for: Adverse Renal Outcomes in Patients With Mesothelioma—A Territory‐Wide Real‐World Data
Source: Cancer Med. 2026 Jan 30;15(2):e71595. doi: 10.1002/cam4.71595 (PMC12856518; doi:10.1002/cam4.71595)
Supplement: Supplementary file 1 — Table S1: Multicollinearity testing of the covariates. Table S2: Risk factors for adverse renal outcomes among patients treated with NCCN recommended treatment. [file CAM4-15-e71595-s001.docx]

**Supplementary Table 1** Multicollinearity testing of the covariates

| **Variables** | **Variance Inflation Factor** |
| --- | --- |
| **Renal progression** |  |
| Age | 1.66 |
| Sex | 1.15 |
| Year of diagnosis | 1.59 |
| Baseline eGFR | 1.34 |
| Hypertension | 1.27 |
| Diabetes mellitus | 1.17 |
| CCI | 1.43 |
| Lines of chemotherapy used | 1.19 |
| Bevacizumab | 1.20 |
| **Upstage of CKD** |  |
| Age | 1.80 |
| Sex | 1.17 |
| Year of diagnosis | 1.76 |
| Baseline eGFR | 1.37 |
| Hypertension | 1.42 |
| Diabetes mellitus | 1.37 |
| CCI | 1.50 |
| Lines of chemotherapy used | 1.49 |
| Third space fluid | 1.32 |
| Ascites | 1.27 |
| Platinum | 1.98 |
| ICI | 1.21 |
| AKI | 1.45 |
| Number of pemetrexed cycles | 1.67 |
| **AKI** |  |
| Age | 1.96 |
| Sex | 1.17 |
| Year of diagnosis | 1.64 |
| Baseline eGFR | 1.43 |
| Hypertension | 1.32 |
| Diabetes mellitus | 1.23 |
| CCI | 1.44 |
| Lines of chemotherapy used | 1.31 |
| Bevacizumab | 1.28 |
| Third space fluid | 11.79 |
| Pleural effusion | 11.81 |
| Number of pemetrexed cycles | 1.37 |
| **Adverse renal outcomes** |  |
| Age | 1.92 |
| Sex | 1.17 |
| Year of diagnosis | 1.65 |
| Baseline eGFR | 1.37 |
| Hypertension | 1.28 |
| Diabetes mellitus | 1.19 |
| CCI | 1.45 |
| Lines of chemotherapy used | 1.77 |
| Ascites | 1.21 |
| Gemcitabine | 1.74 |
| ICI | 1.14 |
| Bevacizumab | 1.14 |

**Supplementary Table 2** Risk factors for adverse renal outcomes among patients treated with NCCN recommended treatment

|  | **OR** | **95% CI** | **p-value** | **aOR^** | **95% CI** | **p-value** |
| --- | --- | --- | --- | --- | --- | --- |
| **Renal progression** | | | | | | |
| Bevacizumab | 11.71 | 2.20 – 62.26 | 0.004 | 11.22 | 1.88 – 89.36 | 0.022* |
| **Upstage of CKD** | | | | | | |
| Ascites | 4.21 | 1.03 – 17.30 | 0.046 | 7.01 | 1.26 – 39.01 | 0.026* |
| ICI | 17.74 | 2.16 – 145.82 | 0.007 | 22.03 | 2.25 – 215.32 | 0.008* |
| AKI | 6.93 | 2.09 – 23.06 | <0.001 | 13.04 | 2.72 – 62.43 | 0.001* |
| Number of lines of cytotoxic chemotherapy | 1.56 | 1.56 – 2.33 | 0.032 | 2.02 | 1.23 – 3.32 | 0.005* |
| Number of pemetrexed cycles | 1.02 | 0.99 – 1.06 | 0.21 | 1.05 | 1.00 – 1.10 | 0.042* |
| **AKI** | | | | | | |
| DM | 3.72 | 1.04 – 13.36 | 0.044 | 16.93 | 1.84 – 156.05 | 0.013* |
| Bevacizumab | 4.93 | 1.11 – 21.89 | 0.013 | 26.37 | 1.67 – 416.30 | 0.020* |
| Third space fluid | 4.67 | 1.29 – 16.94 | 0.019 | 4.62 | 1.03 – 20.80 | 0.046* |
| Pleural effusion | 3.63 | 1.13 – 11.67 | 0.030 | 3.10 | 0.77 – 12.54 | 0.11 |
| Number of pemetrexed cycles | 1.04 | 1.00 – 1.07 | 0.034 | 1.01 | 0.96 – 1.06 | 0.82 |
| **Adverse renal outcomes** | | | | | | |
| DM | 4.60 | 0.93 – 22.72 | 0.061 | 6.26 | 1.10 – 35.38 | 0.038* |
| Ascites | 4.60 | 0.93 – 22.72 | 0.061 | 6.80 | 1.55 – 120.24 | 0.048* |
| ICI | 10.76 | 1.31 – 88.12 | 0.027 | 13.67 | 2.34 – 191.71 | 0.018* |
| Number of lines of cytotoxic chemotherapy | 1.73 | 1.14 – 2.63 | 0.010 | 2.15 | 1.32 – 3.50 | 0.002* |

*: Statistically significant.

^: Adjusted for age, sex, year of diagnosis, presence of hypertension, diabetes mellitus, baseline estimated glomerular filtration rate, lines of chemotherapy and Charlson co-morbidity index.

OR, Odds ratios; aOR, adjusted odds ratios; CI, confidence interval; ICI, immune check-point inhibitors; AKI, acute kidney injury; DM, diabetes mellitus.
